# Informal care provision among male and female working carers: Findings from a Swedish national survey

**DOI:** 10.1371/journal.pone.0263396

**Published:** 2022-03-07

**Authors:** Joana Vicente, Kevin J. McKee, Lennart Magnusson, Pauline Johansson, Björn Ekman, Elizabeth Hanson

**Affiliations:** 1 Faculty of Health and Life Sciences, Department of Health and Caring Sciences, Linnaeus University, Kalmar, Sweden; 2 School of Health and Welfare, Dalarna University, Falun, Sweden; 3 The Swedish Family Care Competence Centre, Kalmar, Sweden; 4 Faculty of Health and Life Sciences, Department of Medicine and Optometry, Linnaeus University, Kalmar, Sweden; 5 Department of Clinical Sciences, Lund University, Malmö, Sweden; University of Bologna, ITALY

## Abstract

**Introduction:**

Informal carers in paid employment–working carers (WKCs)—have complex support needs. However, little is known about WKCs’ pattern of informal care provision, the support they receive, the impact providing care has on their employment, and how these vary between male and female WKCs. This study describes the pattern of informal care provision and received support among Swedish WKCs.

**Research method/Design:**

The study was a cross-sectional questionnaire-based survey of a stratified random sample of the Swedish population aged 18 or over. The questionnaire addressed the type and extent of informal care provided, support received and the impact of care provision on employment. Of the 30,009 people who received the questionnaire, 11,168 (37.3%) responded, providing an analytic sample of 818 (7.32% of respondents) employed or self-employed informal carers.

**Findings:**

A typical Swedish WKC was a middle-aged female, providing weekly or daily care to a non-cohabitant parent, who experiences care as sometimes demanding and receives no formal support as a carer. Female WKCs were more likely than males to care alone and with higher intensity, to report a need for help in meeting their care-recipient’s needs, and to experience care as demanding. Approximately 17% of WKCs reported their employment had been affected due to caring, 40% their ability to work, and 31% their career development opportunities. Female WKCs’ ability to work was affected more than males’, and they were more commonly prevented from applying for work.

**Conclusion:**

Swedish female WKCs compared to males provide more hours of informal care, across more care domains, more often alone. This places them in a challenging situation when combining paid work and care. Greater recognition of the challenges faced by WKCs is required in Sweden and other countries, as are policies to reduce gender inequalities in informal care provision in this group.

## Introduction

Informal care is the term used for a range of different help, support and/or care activities provided on a regular basis by a family member or other unpaid individual to a sick, frail and/or disabled relative or significant other [1,2]. Informal care is a substitute for long-term care [3,4] and approximately 51% of individuals in the EU between 18–64 years old are informal carers [5]. However, despite the number of individuals involved, informal carers receive little formal recognition in many EU countries [6,7]. Proportionately more women than men provide informal care, and women experience several of the negative outcomes associated with being an informal carer to a greater extent than men [8]. In Sweden, as in other countries, a significant growth in the need for long-term care is expected at the same time that formal care services are being reduced [9–11], which has led to an increased need for both informal care and support for informal carers [12–14]. Results from a literature review revealed that across Europe the increased demand for informal care is likely to have a negative impact on women´s participation in the labour market [15]. Informal carers who are in paid employment are referred to as working carers (WKCs) [16], a significant and growing group in society, who due to their dual roles as carers and employees often have complex support needs. If appropriate support is to be provided to WKCs, more research is required on the nature of their caregiving circumstances. This paper presents analyses of data collected via a Swedish national survey, examining the provision of care and received support among male and female WKCs.

### Long-term and informal care policy and practice in Sweden

In Sweden, public expenditure on long-term care ranks among the top three in Europe at 3.5 percent of the gross domestic product (GDP). Long-term care services in Sweden are organized at a local level where municipalities can purchase services from both public and private providers and where long-term care receivers can choose from several care providers [17]. However, even if the long-term care model for frail older and/or disabled people in Sweden is still highly regarded internationally compared to other EU member states [18], demographic ageing trends and economic cutbacks in the municipalities have led to a significant reduction in the number of nursing homes available during the course of the last few decades. In addition, previously generous, universal home help services are increasingly prioritised towards those with the greatest health and care needs living alone [19]. In combination these changes have led to “re-familialization”- in other words, an increasing dependence on family members to provide the bulk of the care to frail older and/or disabled people [20].

As in other countries, organizational changes in Sweden inspired by new public management have led to a reduction in the work satisfaction of formal home care workers and threatened the quality of care for older and disabled people living at home [21].This in turn has led to increased pressures on the family to contribute to the care of their home-dwelling older and disabled relatives. The marketisation of home help services has also led to greater inequalities in care, with older and disabled people with higher formal education more likely to purchase private services themselves, whereas older and disabled people with less formal education are more likely to receive informal care, primarily from an adult daughter [22].

In terms of the legal recognition of informal carers in Sweden, an amendment of the Swedish Social Services Act in 2009 stipulated for the first time that municipalities are obliged to offer support to carers, who are now legally entitled to an assessment of their own needs as carers [23]. However, the exact nature and extent of the support to be provided is not stipulated. An audit of the implementation of the law in 2016 [24] revealed that very few carers were aware of their right to an assessment of their needs, while the exact extent and range of services available varied widely across the country with some municipalities offering more extensive carer support services (including, e.g., information, education and training, group support, individual counselling, respite care services and economic benefits) and a comprehensive assessment of carers’ needs, whereas other municipalities offer minimal support [25]. Recently there has been a call for a national carer strategy to stimulate more comprehensive policies and supports for carers across Sweden [26].

### Working carers and gender

The increased need for informal care in recent decades has disproportionately affected women. Figures from a 2012 Swedish national survey highlighted that 20% of women are informal carers compared to 10% of men [27]. Female carers more often take on long-term care responsibilities with more demanding and intensive care [2], and experience greater burden and stress and lower life satisfaction when compared to male carers [7,28,29].

Sweden is an early adopter of gender-neutral policies with high levels of female workforce participation [30]. The country ranks second globally for the highest proportion of women in the workforce [31] with one of the highest female employment rates in the OECD of 70.7% in 2018, compared to 55.9% in Germany and 58.4% in Denmark [32] respectively. However, even in Sweden, women are overrepresented among low-paid part-time workers and unemployed persons and have lower pension income than men [10,12].

While the health and care needs of both carer and care recipient are major influences on the patterns of informal care, employment characteristics specifically affect WKCs’ patterns of care provision and health [33,34]. WKCs’ type of employment, total hours of work, work schedule and workplace support all affect their ability to provide informal care [33,35]. Further, the combination of unpaid care with paid work can negatively affect both WKCs’ physical and mental health [7,36–38]. Recent studies have revealed that the pattern of care provision and the negative outcomes of informal care differ between male and female WKCs. Female WKCs compared to male WKCs have lower paid working hours, are more likely to reduce their working hours, income and paid hours to undertake care [28,33] and experience higher financial strain from informal care provision [39]. In Sweden, a 2012 national survey revealed that 12% of female WKCs had to reduce their working hours compared to only 9% of their male counterparts [27]. Furthermore, some WKCs have both older parents and adult children with care needs, a situation called sandwich care [40,41]. Female sandwich carers provide more care to their older parents and adult children than do male sandwich carers [42].

Most EU countries lack recognition of and adequate support for WKCs’[43], and work-care reconciliation policies that facilitate and support WKCs´ combination of carer and employee roles are generally of low priority [44]. Sweden has a stated policy goal that all women and men regardless of their social or ethnic background shall have the same access to public support [45,46]. However, WKCs’ employment-related rights remain limited [47] and little is known of what support is offered and/or received by WKCs and whether this varies according to the WKC’s gender. The aims of this study are: a) to describe who Swedish WKCs are, what type of informal care they provide, the impact of providing informal care on their employment and the nature and extent of support they receive; and b) to investigate whether the pattern of WKCs’ informal care provision and received support varies by gender.

## Methods

### Design

The Swedish National Carer Survey was a cross-sectional questionnaire-based population survey of the characteristics of adult informal carers carried out between November 2018 and January 2019.

### Sampling and participants

Statistics Sweden (SCB) randomly selected a sample of 30,009 adults (18 years of age or over) to participate in the survey from the Swedish National Population Registry at the end of July 2018 (total population 8,063,051) from a stratified frame that ensured approximately equal representation from each Swedish region. Selected individuals were sent a questionnaire for self-completion and return. After excluding 365 cases (questionnaire returned, wrong address n = 316; person could not be contacted n = 49) the response rate was 37.3% with 11,168 individuals completing the questionnaire. Reasons for non-response were: questionnaire not returned n = 17,503; participation declined n = 480; prevented from participating n = 120; wrong person answered the questionnaire n = 195; returned questionnaire spoiled n = 86; promised to send in n = 5. The definition of an informal carer provided in the questionnaire was: those who provided care, help or support to a loved one (to someone in the family, or to someone in a close relationship such as a friend, neighbour, or work colleague) in a personal capacity due to their physical or mental illness, disability or old age. The definition excluded care provided in ones role as an employee and care provided by parents to children without special needs. From those individuals indicating that they met the definition of informal carer, those who gave care and support to one or more persons less often than once a month were excluded from the study. The number of working age participants in the sample was 6,432 (57.6% of the total sample of 11,168). Of these, 1,093 met the criteria for being an informal carer (16.9% of the working age sample and 9.78% of the total sample). Of these, the number of employed and self-employed participants was 818 (74.8% of working age carers), and these participants constituted the analytic sample. Estimated to the Swedish 2018 population level, there are 602,926 working carers, that is 7.4% of the total population.

### Procedure

SCB sent the survey questionnaire by surface mail in October 2018, together with an information letter, a postage pre-paid reply envelope and a link to a web-based version of the questionnaire. At the end of October, SCB sent a reminder card and subsequently two additional reminder letters with an additional copy of the questionnaire, the first at the beginning of November and the second at the end of the month. SCB concluded data collection on January 8, 2019. SCB processed the questionnaires, performed quality checks and generated the final dataset, adding in data on participants’ gender and age taken from national registries. The research team further checked and cleaned the anonymized dataset.

### Material

The survey questionnaire was developed by the research team using a previous questionnaire from a 2012 Swedish national survey study as a starting point [27]. For the 2018 questionnaire version, the research team removed some questions from 2012, others were adjusted and some left unchanged. The final questionnaire contained 28 main questions with a variety of response formats including fixed-response options, rating scales and open-ended questions. In this study, questions were selected for analysis that addressed eight areas: sociodemographic characteristics; caregiving characteristics; caregiving context; local authority support; support to care provider, perceived care needs and type of care provided (S1 File).

### Ethical considerations

All participants answered the questionnaire voluntarily and received information on how their personal data would be handled as regulated by the EU data protection Act (2001:99), GDPR (2001:99) and the Ordinance (2001:100) of the Official Statistics and Public and Secrecy Act. All participants provided informed consent for participation in the study when answering the questionnaire. The Regional Ethics Review Board in Linköping approved this study in 2018 (no. 2018/135-31).

### Data analysis

IBM SPSS v.27 (SPSS Inc., Chicago, IL, USA) was used to describe and summarise the characteristics of the sample and to perform univariate and bivariate analyses. Prior to analysis, the response options of some questions were recoded to combine neighbouring response categories as a result of low numbers within categories and/or to facilitate further analysis. As most variables were ordinal or nominal, associations with gender were analysed using Chi-square test or phi coefficient if the variable was dichotomous. During analysis, a p-value of less than 0.05 was the criterion for statistical significance. Due to multiple testing inflating the family-wise error rate each significance test should be considered in the context of the obtained effect size. For all analyses survey weights were applied based on a participant’s gender, age, education, and region to compensate for sampling bias generated by non-random non-response and the sampling frame. Thus, when n is reported hereafter this represents the weighted n, N = 835 for the weighted sample.

## Results

### Working carers’ demographic and caregiving characteristics

The average age of WKCs was 48.6 years, and 56.1% were female. Only 6.4% of female WKCs were self-employed, compared to 20.7% of males. The majority of WKCs (77.5%) cared for one person and were non-cohabitant carers (65.5%). While 43.3% of WKCs provided care at least once a week, 39.6% did so daily. The majority of WKCs (64.5%) cared between 1 and 10 hours a week although 11.2% cared for 130 or more hours per week. Almost half of WKCs experienced care provision as sometimes demanding while for 22.9% care provision was seldom or never demanding. Just under half of the WKCs’ care-recipients were their parents and they were predominantly female (61.9%), with 35% being 80 years of age or older. (see Table 1).

**Table 1:** Sociodemographic variables, caregiving characteristics and context for the total sample of working carers and by gender.

	Total (%) N = 835	Men (%) n = 367	Women (%) n = 469	*p*		
**Sociodemographic**						
Age (years,SD)	48.59 (11.1)	48.67(11.3)	48.53(11.0)	.865^a^		
Employment status (%)				**.000** ^**b**^		
Employed	87.4	79.3	93.6			
Self-employed	12.6	20.7	6.4			
**Caregiving characteristics**						
Person cared for (%)				**.035** ^**c**^		
Husband/wife/partner	10.5	11.1	10.0			
Child	25.4	23.1	27.2			
Parent	47.9	51.9	44.8			
Sibling, relative	11.2	8.1	13.7			
Legal guardian, neighbour, acquaintance	5.0	5.8	4.3			
Number of care-recipients (%)				.175^**c**^		
One person	77.5	80.1	75.4			
Two people	17.1	16.3	17.7			
Three people	2.0	1.4	2.6			
More than three people	3.4	2.2	4.3			
Frequency of care (%)					.232^**c**^
Everyday	39.6	38.1	40.8			
At least once a week	43.3	42.3	44.1			
At least once a month	17.1	19.6	15.1			
Intensity of care (%)				**.002** ^**c**^		
Less than 1 hour/week	9.3	13.2	6.2			
1–10 hours/week	64.5	62.6	65.8			
11–29 hours/week	15.0	13.2	16.3			
30–59 hours/week	7.3	8.6	6.5			
60 or more hours/week	3.9	2.3	5.2			
Experience care as demanding (%)				**.000** ^**c**^		
Always/Almost always	9.5	8.4	10.3			
Often	18.3	10.8	23.9			
Sometimes	49.3	49.4	49.3			
Seldom/never	22.9	31.4	16.7			
Caregiving context					
Care-recipient´s age (%)				.470^**c**^		
< 18 years	15.9	14.9	16.7			
18–29 years	11.8	11.6	12.0			
30–44 years	6.3	5.8	6.7			
45–64 years	14	14.9	13.3			
65–79 years	17	14.9	18.7			
> 80 years	35	38.0	32.6			
Care-recipient´s gender (%)				.558^b^		
Women	61.9	63.0	61.0			
Men	38.1	37.0	39.0			
Co-residence (%)				.897^b^		
Yes	34.6	34.3	34.8			
No	65.4	65.7	65.2			

Note: n for analysis varies between n = 835 and n = 812 due to internal missing data; significant p values are presented in bold.

^a^ Independent samples t-test

^b^ phi coefficient

^c^ Chi-square test.

### Support provided to the care-recipient

**Fig 1:**
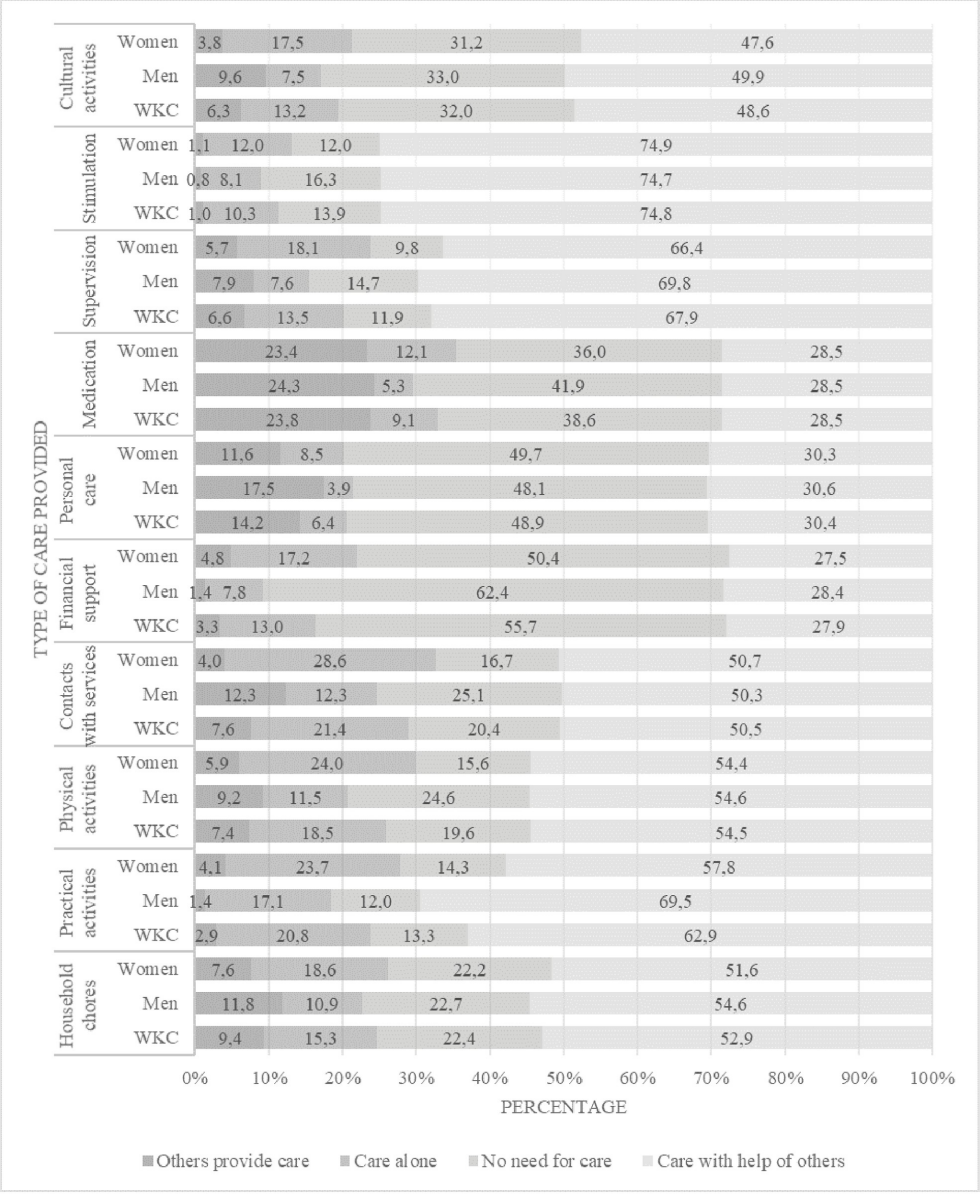
Type of care provided for ten domains of care for the total samples of working carers and by gender. Note: n for analysis varies between n = 817 and n = 797 due to internal missing data.

For each of the 10 domains of care, respondents were asked whether a) they provided support to their care-recipient alone; b) they provided support with the help of others; c) support to the care-recipient was provided only by others; or d) the care-recipient did not need support (see Fig 1). For most care domains, more than half of WKCs provided care with the help of others: stimulation (74.8%), supervision (67.9%), practical activities (62.9%), physical activities (54.5%), household chores (52.9%) and contact with services (50.5%). For three care domains, the largest proportion of WKCs reported that their care-recipient did not need care: financial support (55.7%), personal care (48.9%) and medication (38.6%).

Almost three out of five of the WKCs’ care-recipients received support from their local authority. Nearly equal proportions of WKCs felt their care-recipient’s needs were met (47.2%) as felt that they would like more help to meet their needs (47.1%). (Table 2).

**Table 2:** Local authority support to care-recipient and carer and carer perceived needs of care-recipient for the total sample of working carers and by gender.

	Total (%) N = 835	Men, (%) n = 367	Women, (%) n = 469	*p*
**Local authority Support**				
Local authority support to care-recipient (%)				.262^a^
Yes	58.9	57.7	59.9	
No	32.3	31.7	32.8	
Don’t know	8.8	10.6	7.4	
Local authority support to carer (%)				.090 ^b^
Yes	16.1	18.6	14.2	
No	83.9	81.4	85.8	
**Perceived care needs of care-recipient (%)**				**.019** ^**a**^
All care-recipient’s needs are met	47.2	52.5	43	
Will provide more support to care-recipient	5.8	5.9	5.7	
Would like more help to meet care-recipient’s needs	47.1	41.6	51.3	

Note: n for analysis varies between n = 824 and n = 816 due to internal missing data; significant p values are presented in bold.

^a^ Chi-square test

^b^ phi coefficient.

### Working carers’ receipt of carer support

**Fig 2:**
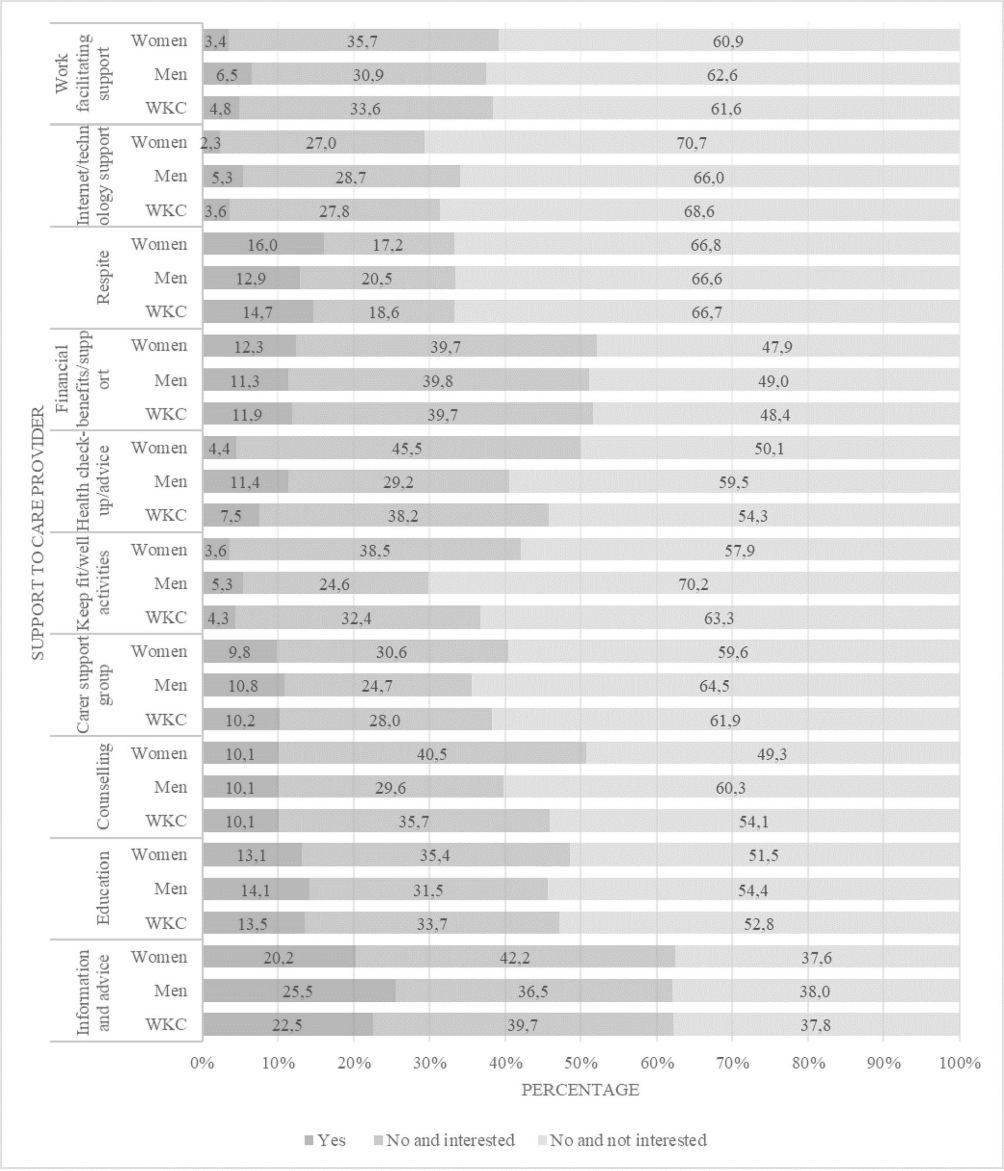
Receipt of ten types of local authority carer support for the total sample of working carers and by gender. Note: n for analysis varies between n = 789 and n = 769 due to internal missing data.

Only 16.1% of WKCs reported that they received carer support from their local authority (see Table 2). The questionnaire listed ten different types of support currently available for carers (see Fig 2). Respondents indicated for each type of support whether: a) they received or had been offered the support; b) they had not received/been offered the support but were interested in it; or c) they had not received/been offered the support and were not interested in it (see Supporting information S1). Across the 10 types of support, between 37.8% and 68.6% of WKCs indicated they had not received/been offered the support and were not interested in it. By comparison, 18.6%-39.7% of WKCs indicated they had not received/been offered the support but were interested in it. Information and advice was the most common type of support received by/offered to WKCs (22.5%) followed by respite care (14.7%), education (13.5%) for carers, and financial benefits (11.9%).

### Associations between patterns of care and gender

Analyses indicated several significant associations between WKCs’ gender and their sociodemographic and caregiving characteristics. From Table 1, the association between gender and employment status was significant (φ (1) = -21, p < .001), with male WKCs disproportionately self-employed (20.7%) and female WKCs disproportionately employed (93.6%).Female WKCs compared with males were more likely to care for a child (women, 27.2%; men, 23.1%) or sibling/other relative (women, 13.7%; men 8.1%), while male WKCs compared with female were more likely to care for a parent (men, 51.9%; women, 44.8%;χ2 (4) = 10.37, p = .035). There was a significant association between gender and intensity of care (χ2 (4) = 17.54, p = .002), where the clearest difference between men and women was that male WKCs were disproportionately represented in the lowest category of caregiving intensity (less than one hour per week) compared with females (men:13.3%; women 6.2%). There was also a higher representation of women compared to men in the highest category (60 hours a week or more; women, 5.2%; men 2.3%). Providing care was experienced as often demanding by 23.9% of female WKCs compared with 10.8% of male WKCs, while 31.4% of male WKCS found caring seldom or never demanding compared with 16.7% of female WKCs (χ2 (3) = 38.23, p < .001). Gender was significantly associated with the pattern of WKCs’ care provision (see Fig 1). The clearest variation between men and women was found in the care alone response category, where there was proportionately more female WKCs and fewer male WKCs for nine out of the ten care domains,: household chores (χ2 (3) = 11.75, p = .008); practical activities (χ2 (3) = 14.45, p = .002); physical activities (χ2 (3) = 28.16, p < .001); contacts with services (χ2 (3) = 50.04, p < .001); financial support (χ2 (3) = 25.93, p < .001); personal care (χ2 (3) = 11.54, p = .009); medication (χ2 (3) = 12.12, p = .007); supervision (χ2 (3) = 21.99, p < .001) and cultural activities (χ2 (3) = 25.61, p < .001). The majority (52.5%) of male WKCs indicated that their care-recipient´s needs were being met, whereas the majority (51.3%) of female WKCs indicated they would like more help to meet their care recipient´s needs (χ2 (2) = 7.89, p = .019) (see Table 2).

While there was no significant association between gender and receipt of local authority carer support to WKCs (see Table 2), significant associations were found for three specific forms of carer support: keep fit/well activities, health check-up/advice, and counselling (see Fig 2). The same pattern of responses was apparent for keep fit/well activities and health check-up/advice: male compared with female WKCs had disproportionately received/been offered the support, or were not interested in the support; correspondingly, female compared with male WKCs were disproportionately interested in the support (keep fit/well activities: χ2 (2) = 17.28, p < .001; health check-up/advice, χ2 (2) = 28.55, p < .001). With regard to counselling support, 10.1% of both female and male WKCs had received/been offered counselling support. However, among those who had not received/been offered counselling, 40.5% of women were interested in the support compared with 29.6% of men (χ2 (2) = 10.86, p = .004) (see Fig 2).

### Combining paid work and informal care

Approximately 17% of WKCs indicated that their employment status had been affected due to their caring activities, with no significant association with gender. Of those WKCs who indicated that their employment status had been affected, 30.4% had reduced their work/study hours by at least 50% (see Table 3). Female compared with male WKCs disproportionately reduced their hours by 50% (women, 21.3%; men,5.5%), with male compared with female WKCs more likely to reduce their hours by greater than 50% or less than 50% (χ2(2) = 6.99, p = .030).

**Table 3:** Impact of providing care and support on work/study among working carers: Associations with gender.

Impact	Total (%)	Men (%)	Women (%)	*p*
**Employment has been affected (n = 135)**				**.030** ^**a**^
Reduced work/study hours >50%	15.6	20.0	12.4	
Reduced work/study hours = 50%	14.8	5.5	21.3	
Reduced work/study hours <50%	69.6	74.5	66.3	
**Ability to work/study decreased (n = 331)**				**.007** ^**a**^
Decreased about 10%	52.6	60.7	46.3	
Decreased by 10–25%	26.9	18.6	33.3	
Decreased by more than 25%	20.5	20.7	20.4	
**Opportunities to develop career/studies affected** ^*^ **(n = 261)**				
Had to leave work/studies	5.4	2.8	7.1	.113^b^
Not able to give time to work/studies†	77.7	85.8	72.1	**.009** ^b^
Only take temporary/short-term work/studies	2.7	4.7	1.3	.099^c^
Prevented from applying to work/study	5.0	0.9	7.7	**.013** ^b^
Opportunities affected in other way	11.5	9.4	12.9	.388^b^

Note

*percentages responding ‘yes’ to each question presented

^†^ n = 260

^a^ Chi-square test

^b^ phi coefficient

^c^Fisher’s exact test; significant p values are presented in bold.

Approximately 40% of WKCs indicated that their ability to work/study had decreased due to their caring activities, with no significant association with gender. Of those WKCs who indicated that their ability to work/study had decreased, just over half indicated their ability had decreased by around 10% (see Table 3). Female compared with male WKCs disproportionately indicated that their ability to work/study had decreased by 10%-25%, (women, 33.3%; men, 18.6%), with male compared with female WKCs more likely to indicate that their ability to work/study had decreased by about 10% (men, 60.7%; women, 46.3%; χ2(2) = 9.80, p = .007).

Approximately 31% of WKCs indicated that opportunities to develop their career/studies had been affected due to their caring activities, with no significant association with gender. Of those WKCs who indicated their opportunities to develop their career/studies had been affected, 5.4% indicated they had had to leave their work/studies altogether, 77.7% indicated they had not been able to give the time to their work/studies that they wanted, 2.7% indicated they had only been able to take on temporary or short-term work/studies, 5.0% indicated they had been prevented from applying for work/studies, while just over 11% indicated that providing care and support had affected their opportunities to develop their career/studies in other ways (see Table 3). A higher proportion of male than female WKCs indicated they had not been able to give the time to their work/studies that they wanted (85.8% vs 72.1%, φ (1) = -.163, p = .009), while a higher proportion of female than male WKCs indicated they had been prevented from applying for work/studies (7.7% vs 0.9%, φ(1) = .153, p = .013).

## Discussion

The aims of this study were to describe the pattern of informal care provision and received support among Swedish WKCs and to investigate whether the pattern varied by gender. While there is a growing number of studies on the importance of WKCs as informal care providers and employed female carers in particular [39,48], our study adds valuable information as relatively little is known about Swedish WKCs. Our findings indicate that gender does play a role in the type of care provided, the impact of care on employment and the type of carer support received. Female WKCs cared for 11 hours or more per week, more often cared alone, more often experienced care as demanding, had more difficulty to apply for a job or continue their studies and were less likely to have received/ been offered two types of carer support than their male counterparts. Below we consider the picture that emerges from our findings of the pattern of informal care provision and received support among WKCs in Sweden, with a particular focus on gender.

### Gender and working carers’ patterns of care

Our findings indicate that a ´typical´ Swedish WKC can be characterised as a middle-aged female caring for a non- co-habitant parent on a weekly or daily basis. This picture corresponds to that presented in a previous national study conducted in Sweden [27].

Previous research has indicated that the provision of high intensity care has detrimental effects on the carer’s physical and mental health [12,49] and women more often experience informal care as demanding compared to men [7,28]. In our study, while the majority of WKCs provided care between 1–10 hours per week, men compared with women were more likely to provide care for less than 1 hour a week, while women were more likely than men to provide 60 hours of care or more a week. Just over 30% of female WKCs found providing care to be often to always demanding, compared with just under 20% of males WKCs. Previous evidence supports this distribution of intensive informal care provision [50–52], even in Sweden, where women provide higher intensity informal care (mean 5,8 hours of care per week) compared to men (mean 3,8 weekly hours provided) [27].

In our study female WKCs more often than males provided care alone across most care domains such as personal care, physical activities, medication, and contact with services. These findings correspond with the existing literature on the role of female informal carers in providing personal care and more complex care activities [53,54], compared to male informal carers who, more commonly, support the care-receiver´s instrumental activities and provide financial support[55,56].

### Gender and working carer support

Combining work and care is associated with several challenges that require adequate solutions for WKCs support [57]. Previous literature has described forms of carer support for WKC such as technology-based solutions and workplace measures [50,58]. However, little is known about how much carer support is provided to WKCs in Sweden. In our study, out of ten types of support for carers, only one information and advice, was received by/offered to over 20% of WKCs. This finding of a very low level of carer support for WKCs is surprising in the context of the amendment to the Swedish Social Act (2009) which states that municipalities are obliged to offer support to carers and that carers have a right to an assessment of their own needs [23].

Furthermore, for 9 out of the 10 types of carer support considered, between a third and two-thirds of WKCs had not been offered/received the support and were not interested in it. One explanation for this finding could be that as nearly 75% of WKCs provided low or medium intensity care and, depending on the care domain, between 27.5% and 74.8% provided care with the help of others, the support they received as carers was sufficient for their own needs. An alternative explanation is that some WKCs were not aware of the exact nature of the various types of carer support considered, and so were unable to make an informed decision as to their personal relevance and value. Nevertheless, a substantial minority of WKCs were interested in some types of carer support that they had not received/ been offered, particularly financial support (39.7%), information and advice (39.7%) and a health check-up (38.2%). Female WKCs were in general more interested in receiving carer support than male WKCs. In addition, just over 47% of WKCs felt that they would like more help to meet the needs of their care-recipient, the proportion greater among female compared with male WKCs. Taken together, it seems that a considerable proportion of WKCs (between 18.6% and 39.7%), particularly female WKCs, wished more support for themselves as carers and to mee the needs of the care-recipient, and that a starting point would be to improve the information about the types of support available to them.

### Gender and working carers´ s employment

In a UK study analysing the cost of providing formal care services to replace the informal care provided by WKCs, Pickard (2019) found that in terms of public expenditure the replacement care services cost less than did the loss of WKCs to the labour market [59]. Further, a recent cost-analysis of informal care in Sweden, highlighted that the cost of informal care provided by adults is around SEK 152 billion per year, with the larger costs related to carers’ reduced paid work hours [60]. These findings emphasize the societal importance of maintaining WKCs in the labour market. The 2012 Swedish national survey, mentioned previously, estimated that 100,000 people of working age (18 to 64 years old) found it difficult to combine paid work with informal care [40]. In our study, more than one out of six WKCs indicated that their employment status had been affected, more than two out of five WKCs experienced a decreased ability to work/study due to their caring situation and just under a third indicated that their opportunities to develop their career/studies had been affected. Taken together, the evidence suggests that the challenge of combining paid work and informal care has not been satisfactorily addressed since the earlier survey.

While our results indicate that employment status, ability to work, and career opportunities affect similar numbers of male and female WKCs, within those WKCs affected, gender variations were found on the nature and level of effect. That is, in WKCs whose employment status had been affected, males compared to females tended to reduce working hours by larger or smaller amounts. In those WKCs who indicated their ability to work/study had decreased, the decrease was greater among females compared to males. Among WKCs whose opportunities to develop their careers/studies were affected, males to a greater extent than females were unable to give the time they wanted to their career/studies, while females to a greater extent than males were unable to apply for work/study. These findings are generally in line with other studies concerning the gendered limitations for informal carers to participate in paid work. Female informal carers in Sweden are known to take more sick leave and experience greater challenges when applying for a job compared to male informal carers [27] with as many as 90,000 women and 50,000 men, between 45–66 years, having to reduce their working hours, quit the labour market or take early retirement due to their caring activities [61].

From a family perspective, one interpretation of the gender associations with paid work found in this study is that when the need for informal care provision starts or increases, the family member who is most likely to stop work or change employment status to meet that need is the one earning less money and/or with unstable employment condition such as part-time work. This person will most often be the adult female family member [8,10,33]. Literature confirms that high intensity carers experience increased effects of caregiving while in employment [62,63]. Our findings, that female compared to male WKCs tend to provide care for more hours per week, experience care as more demanding, more often care alone and receive low levels of carer support, provide the context for understanding the challenges female WKCs face in combining paid work and care.

### The international context

It would be anticipated that the picture of informal care provided by our study might be comparable to those found in other countries with relatively strong welfare states. This would seem to be the case. A study of census data from the United Kingdom found that a greater proportion of women than men were informal carers and that female informal carers provided more care than male informal carers [64]. A recent study from the Netherlands found that informal carers were predominantly women, the majority aged between 45–64 years, providing care to mostly close family members. The study concluded that policies to support informal carers should focus on the combination of work and care [65].

It would also be expected that there would be considerable differences in the picture of informal care presented when comparing Sweden with countries with a family-oriented welfare model, such as southern and eastern European member states. In such countries, there is a normative expectation that care and support for older and disabled people should be primarily provided by family members, predominantly females [63]. However, while the level of informal care provided in such countries is greater than that in countries where there is an expectation that the state should be the primary source of support for older and disabled people, such as Sweden, our study findings would suggest that the patterns of gendered care provision are somewhat similar.

Across Europe, most working age informal carers combine care with part-time work, and research indicates that older women are less often employed regardless of the intensity of care they provide [18]. Findings from studies in other European countries indicate that informal care has a negative impact on carers’ employment, with younger women who provide more care in risk of lower income due to reduced working hours [18,66]. Meanwhile, several international studies show that female WKCs are more prone to reduce and limit their participation in paid work compared to men [2,33,67]. All of the above findings resonate with those in our study. Thus, despite the relative generosity of the Swedish welfare state, and Sweden’s pre-eminence as a gender-neutral country, our study findings reflect the general pattern across Europe: women compared to men undertake more demanding levels of informal care and experience more negative outcomes; and female WKCs’ ability to work and apply for employment is compromised more than male WKCs.

A recent European study showed that women with lower education were more likely to provide high intensity care than men [68] and even if our study did not approach the differences between educational background within WKCs, our results show that more women than men engaged in long weekly care hours and reported a decreased ability to work or study due to informal care provision, which might affect their educational opportunities in the long run.

### The policy context

A recent study found that the lack of supportive policies and actual support for informal carers within the European Union increases the negative consequences of informal care provision such as unemployment [69], while a recent European report concluded that better support and policies are needed for informal carers across the EU [18]. Sweden was one of the first countries to implement gender-equality policies, with policies such as individual income taxation, the development and expansion of public childcare, and access to gender neutral parental leave in place since the 1970s [70]. However, while Sweden is ranked second globally in terms of having the highest proportion of women in the paid workforce [20] and was an early adopter of gender-neutral policies for the reduction of gender inequalities and the inclusion of women in the labour market [19], our study indicates that female WKCs compared to their male counterparts face greater challenges with regards to combining employment or career development with providing care. These findings raise concerns for the future sustainability of the current informal care pattern. As females provide proportionately more informal care than men, where female WKCs have to choose between care provision or employment, gaps in informal care may occur [71], or, more likely they may withdraw from the labour market due to their employment being more unstable or providing a lower income than that of male family members [33,67]. A further consideration is the fact that while male WKCs contribute less, and less intense, informal care than female WKCs, other aspects of care provision also vary by gender. Thus, policies for the reconciliation of informal care and employment must be sensitive to these gender variations and the different care roles and positions male and female WKCs might occupy.

The impact of informal care provision on WKCs and subsequently on society confirms the need for targeted policies that ensure the recognition and support of this particular group of informal carers in Sweden. Policies that build a carer-friendly society are requested but even today WKCs are more invisible in Sweden than in e.g., the United Kingdom, with Swedish policy documents focusing on informal carers in general and lacking specific mention of WKCs [46].

Results from the Eurobarometer 76.2 indicated that measures to support the combination of paid work and care for older people are highlighted by European citizens as a way for governments to support carers. Thirty seven percent of participants from Finland, Austria and Sweden, suggested that carers should be allowed to work flexible hours, while 31% thought that carers should have the right to work part-time [72]. A way to support WKCs and allow them to maintain their employment is to recognise employees´ care responsibilities and implement measures that support carers in their workplace [57,73]. Within the EU, employment policies should include measures for informal carers who are in employment, particularly female carers [44]. In addition, as mentioned in the 22^nd^ UNECE Policy Brief on Ageing, access to a period of leave in order to provide care and flexible working arrangements are good examples of policies that can facilitate the reconciliation of employment, informal care, and personal life [74]. Moreover, policies that support work-life balance and at the same time address women’s underrepresentation in the labour market, have to be implemented in all EU countries by August 2022 as part of the work-life balance directive targeting WKCs [75].

### Study strengths and weaknesses

Some study limitations need to be considered alongside the interpretation of the results. The response rate for our study was only 37.3%, although low response rates are not an uncommon problem in postal survey studies [76]. However, a strength of our study is that the large, national, random sample combined with the weights used to compensate for the low response rate and any sampling and non-response bias, ensured that our sample is highly representative of the population. It is important to consider however that there is no consensual definition of an informal carer, and that the informal carers in our study were identified by a combination of a) participants recognising the definition of informal care we provided as a reflection of their own position, and b) our inclusion criteria. As such, our study findings should be generalised with caution to other studies of informal care which may apply a different definition of informal care or use different inclusion criteria. A further limitation of our study is that to cover many topics within a relatively brief questionnaire, it was necessary to use largely single-item measures and no multi-item validated instruments or scales. Our study comprises self-report data, and there is always a need to reflect on the reliability of such data. More pertinently, most studies of informal care, including ours, collect data from the carer rather than the care-recipient, and very few studies have reported comparisons of data collected from both actors [77]. Those that have indicate discrepancies between the actors in e.g., estimates of the level of care provided and the number of care domains for which support is required [78]. Thus, it should be remembered that the picture of informal care provided by the carer is not necessarily the same as that which would be obtained from the care-recipient [79].

Our study was cross-sectional and primarily descriptive, and therefore no causality can be attributed to the associations found in our analyses. Men and women vary on many characteristics—such as education level, health status, and, with regard to informal care characteristics, the intensity of care and the breadth of care tasks undertaken—that might be argued to have greater explanatory power for our associations between gender and e.g., employment status and ability to work. However, an explanatory analysis of a large number of potentially influential and often co-varying factors is complex and requires grounding in theory and empirical evidence. Such an analysis was felt to be beyond the scope of our study, the purpose of which was rather to contribute towards the necessary accumulation of evidence required to guide future explanatory analyses.

## Conclusion

This is one of relatively few studies to explore the association between WKCs’ gender and their informal care provision. WKCs make up a substantial part of those informal carers of working age in Europe today. Female compared to male WKCs in Sweden provide care more often, more intensively more often alone and find it more demanding, placing them in a more challenging situation compared to their male counterparts when attempting to combine paid work and care. Given that Sweden is regarded as one of the most gender-equal countries in the world, we reflect that the challenges faced by female compared to male WKCs in other countries might be even greater. Wider acknowledgement of gender differences in the informal care provided and support received by WKCs is required if appropriate gender-adjusted policies and support measures are to be developed in the future. Further research is needed on the impact of informal care provision on WKCs both in Sweden and other countries, and on the evaluation of policies to reduce gendered inequalities in the pattern of informal care provision in this group.

## Supporting information

S1 File Variable categories for the survey. This table presents the original and recoded variable for the survey.
(DOCX)
