# Supplementary material for: Informal care provision among male and female working carers: Findings from a Swedish national survey
Source: PLoS One. 2022 Mar 7;17(3):e0263396. doi: 10.1371/journal.pone.0263396 (PMC8901065; doi:10.1371/journal.pone.0263396)
Supplement: S1 File — This table presents the original and recoded variable for the survey. (DOCX) [file pone.0263396.s001.docx]

| **Sociodemographic variables** | |
| --- | --- |
| 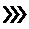 | ***“When were you born”*** *Response options:* Year |
| 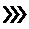 | ***“What is your current main employment”***  *Response options:* ‘Work as an employee’; ‘Self-employed’; ‘Student, intern’; ‘On parental leave’; ‘On leave’; ‘In labour market measures’; ‘Unemployed’; ‘Retired /old age pensioner’; Sickness or activity allowance (early retirement, sickness benefit)´; ´Long-term sick leave (more than 3 months)´; ´Housewife/husband (not pensioner) and ´Other´ |
| 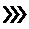 | ***“Who is the person you care and support the most?”*** *Response options:* ‘Husband/wife/partner’; ‘Child’; ‘Parent’; ‘Sibling, relative’ and ‘Legal guardian, neighbour, acquaintance’ |
| **Caregiving characteristics** | |
| 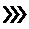 | ***“Do you regularly (i.e. not occasionally or temporarily) care and/or support) someone or several people with their daily activities, personal care or in other ways due to their physical or mental illness, disability or age?”***  *Response options:* ‘Yes, one person’; ‘Yes, two people’; ‘Yes three people’; ‘Yes, more than three people’ and ‘No’ |
| 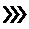 | ***“Think about the care and support you give in total (to one or more people), how often does that occur?”*** *Response options:* ‘Everyday’; ‘At least every week’; ‘At least once a month’ and ‘Less often than once a month’ |
| 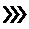 | ***“How many hours a week (day and night) on average do you provide care and support?”***  *Response options:* ‘Less than 1 hour’; ‘1-10 hours’; ‘11-29 hours’; ’30-59 hours’ and ’60 hours or more’  *Comments:* The recoded answers included: ‘Low intensity WkCs (LI)’ (included answer ‘Less than 1 hour’); ‘Medium intensity WkCs (MI)’ (included answer ‘1-10 hours’) and ‘High intensity WkCs (HI)’ (merged answers; ‘11-29 hours’; ’30-59 hours’ and ’60 hours or more’). |
| **Caregiving context** | |
| 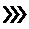 | ***“How old is the person you care and support?”*** *Response options:* ‘Under 18’; ‘18-29; ’30-44’;’45-64’;’65-79’ and ’80 or older’ |
| 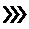 | ***“What gender is the person you care and support most?”***  *Response options:* ‘Man’ and ‘Women’ |
| 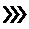 | ***“Where do you live in relation to the person you care and support?”***  *Response options:* ‘In the same household’; ‘In different households but in the same building’; ‘Within walking distance’; ’Not within walking distance, but less than 30 minutes one-way travel’; ‘Between 30 minutes and one hour´s one-way travel’; ‘ Between one-and three-hours one-way travel’;’ Between three- and five-hours one-way travel’ and ‘Over five hours one-way travel’  *Comments:* This variable generated a new dichotomous variable with the categories ‘Yes’ and ‘No’. The ‘Yes’ category included answers ‘In the same household’. The ‘No’ category merged the answers ‘In different households but in the same building’; ‘Within walking distance’; ’Not within walking distance, but less than 30 minutes one-way travel’; ‘Between 30 minutes and one hour´s one-way travel’; ‘ Between one-and three-hours one-way travel’;’ Between three- and five-hours one-way travel’ and ‘Over five hours one-way travel’. |
| **Council support** | |
| 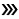 | ***“Has the person or persons to whom you provide care and support been offered health and/or social care services from the municipality?”***  *Response options:* ‘Yes, and generally I have been pleased with the health and social care services provided’; ‘Yes, and generally I have been dissatisfied with the health and social care services provided’; ‘Yes, the person / persons have been offered health and/or social care services but have declined’; ‘No, the person / persons have applied for health and/or social care services but their application has been rejected’; ‘No, the person / persons have not been offered health and/or social care services, but we know who we should contact if necessary’; ‘No, the person / persons have not been offered health and/or social care services and we do not know who we should contact if necessary’ and ‘Don’t know’  *Comments:* This new variable included three response categories ‘Yes’, ‘No’ and ‘Don´t know’. The ‘Yes’ categories merged the original answers: ‘Yes, and generally I have been pleased with the health and social care services provided’; ‘Yes, and generally I have been dissatisfied with the health and social care services provided’; ‘Yes, the person / persons have been offered health and/or social care services but have declined’. The ‘No’ category merged the original answers: ‘No, the person / persons have applied for health and/or social care services but their application has been rejected’; ‘No, the person / persons have not been offered health and/or social care services, but we know who we should contact if necessary’ and ‘No, the person / persons have not been offered health and/or social care services and we do not know who we should contact if necessary’. Finally, the ‘Don’t know’ category included the answer; ‘Don’t know’. |
| 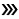 | ***“Have you been offered support from the municipality to assist you personally in your role as a carer (e.g. information, advice, counselling, and information about support groups for carers, education, or other support)?”*** *Response options:* ‘Yes, and I have been generally satisfied with the support I have received’; ‘Yes, and I have been generally dissatisfied with the support I have received’; ‘Yes, I have been offered support but I declined’; ‘No, I have applied for support but my application was rejected’; ‘No, I have not been offered support, but I know who to contact if necessary’; ‘No, I have not been offered support, and ‘I don’t know who to contact if necessary’  *Comments:* This new dichotomous variable included two response categories ‘Yes’ and ‘No’. The ‘Yes’ category merged the original answers: ‘Yes, and I have been generally satisfied with the support I have received’; ‘Yes, and I have been generally dissatisfied with the support I have received’ and ‘Yes, I have been offered support but I declined’. The ‘No’ category merged the original answers: ‘No, I have applied for support but my application was rejected’; ‘No, I have not been offered support, but I know who to contact if necessary’ and ‘No, I have not been offered support, and I don’t know who to contact if necessary’. |
| **Support to care provider** | |
| 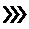 | ***“Below is a list of different types of support for carers. Indicate for each type of support the extent to which it applies to you.”***  ***Subcategories: 1.“Information and advice”, 2.“Education”, 3.“Counselling”, 4.“Carer Support group”, 5.“Keep-fit/well activities (Nordic walking, water-gymnastics, massage or similar)”, 6.“Health check-up and health advice”, 7.“Financial benefits or financial support”, 8.“Respite (temporary formal care for the person(s) you care and support, to relieve you of your care responsibilities, whether delivered at home, via day care, or in a residential care facility”, 9.“Support via modern technology or the Internet (e.g. web information, video conferencing, Internet groups, GPS system)”, 10.“Support that facilitates work (Possibility of distance work, flexible working hours, communication with work managers via the internet or similar)”***  *Response options:* ‘Yes, I have been offered / received this kind of support’; ‘No, I have not been offered / received this kind of support but would like this kind of support’ and ‘No and I'm not interested in this kind of support’ |
| **Perceived care needs** | |
| 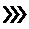 | ***“Do you think that the care and support that this person receives (i.e. from you, family / friends, municipality or other actors) is sufficient to meet his/her needs?”*** *Response options:* ‘Yes, all his/her needs for care and support are currently met’; ‘No, and I would like to contribute more personally’; ‘No, and I would like more help from other family members / friends’; ‘No, and I would like more help from the municipality or equivalent’ and ‘No, and I would like more help from both family / friends and the municipality or equivalent’ |
| **Type of care provided** | |
| 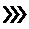 | ***“Here are some examples of activities and other things with which the person you care and support can need help.”***  ***Subcategories: 1.“Shopping, cleaning, washing, cooking, etc.”, 2.“Practical activities such as transport, fixing things in the home and / or garden, bank and postal errands, and other services”, 3.“Physical activity, such as being encouraged to take physical exercise or being accompanied on walks”, 4.“Contacts with health and/or social care services (medical appointments, contact with home help etc.)”, 5.” Financial support / everyday expenses”, 6.“Personal care (such as hygiene, dressing and undressing, help with eating)”, 7.“Medicines and treatment (e.g., giving out medicines, wound dressing, injections)”, 8. “Supervision (looking in on, reminding, motivating, support on distance,)”, 9. “Social Relationships/companionship (togetherness, stimulation, entertainment)” and 10. “Cultural activities, such as being encouraged to engage in cultural activities or being accompanied to cultural activities”***  *Response options:* ‘I am alone in assisting with *subcategory’,* ‘I assist with *subcategory* with the help of other family members / friends only’, ‘I assist with *subcategory* .with support from the municipality or equivalent only’, ‘I assist with *subcategory*, with the help of both other family members / friends and the municipality or equivalent’, ‘Only other people assist with  *subcategory*, not myself’  ‘He/she does not need help with *subcategory*’  *Comments:* A new variable for the type of care provided included four response categories. The new categories were ‘No need to care’ (included the original sixth answer category in all sub-question); ‘Care alone’ (included the original first answer category in all sub-questions); ‘Care with help of others’ (included the original second, third and fourth answer categories in all sub-questions) and ‘Other provide care’ (included the fifth answer category in all sub-questions). |
| ***S1 file -***Variable categories for the population survey (“Questions”, ‘response options’ and comments). | |
